# Mcl-1 Ubiquitination: Unique Regulation of an Essential Survival Protein

**DOI:** 10.3390/cells3020418

**Published:** 2014-05-08

**Authors:** Barbara Mojsa, Iréna Lassot, Solange Desagher

**Affiliations:** 1 Institut de Génétique Moléculaire de Montpellier UMR 5535 CNRS, 1919 route de Mende, 34293 Montpellier cedex 5, France; E-Mails: barbara.mojsa@igmm.cnrs.fr (B.M.); irena.lassot@igmm.cnrs.fr (I.L.); 2 Université Montpellier 2, Place Eugène Bataillon, 34095 Montpellier cedex 5, France; 3 Université Montpellier 1, 5 boulevard Henri IV, 34967 Montpellier cedex 2 France

**Keywords:** Mcl-1, ubiquitin, proteasome, apoptosis, phosphorylation, E3 ubiquitin-ligase, deubiquitinase

## Abstract

Mcl-1 is an anti-apoptotic protein of the Bcl-2 family that is essential for the survival of multiple cell lineages and that is highly amplified in human cancer. Under physiological conditions, Mcl-1 expression is tightly regulated at multiple levels, involving transcriptional, post-transcriptional and post-translational processes. Ubiquitination of Mcl-1, that targets it for proteasomal degradation, allows for rapid elimination of the protein and triggering of cell death, in response to various cellular events. In the last decade, a number of studies have elucidated different pathways controlling Mcl-1 ubiquitination and degradation. Four different E3 ubiquitin-ligases (e.g., Mule, SCF^β-TrCP^, SCF^Fbw7^ and Trim17) and one deubiquitinase (e.g., USP9X), that respectively mediate and oppose Mcl-1 ubiquitination, have been formerly identified. The interaction between Mule and Mcl-1 can be modulated by other Bcl-2 family proteins, while recognition of Mcl-1 by the other E3 ubiquitin-ligases and deubiquitinase is influenced by phosphorylation of specific residues in Mcl-1. The protein kinases and E3 ubiquitin-ligases that are involved in the regulation of Mcl-1 stability vary depending on the cellular context, highlighting the complexity and pivotal role of Mcl-1 regulation. In this review, we attempt to recapitulate progress in understanding Mcl-1 regulation by the ubiquitin-proteasome system.

## 1. Introduction

Apoptosis is an evolutionarily conserved form of programed cell death that plays a crucial role in development, tissue homeostasis and defense against infected and potentially harmful cells. Alterations in apoptotic pathways are involved in many human disorders: insufficient apoptosis is necessary for tumorigenesis and favors autoimmunity, while increased apoptosis is evident in neurodegenerative diseases, AIDS and infertility [1]. Apoptosis results from the activation of a family of cysteine proteases, named caspases which are responsible for the dismantling of the cell and the characteristic morphological changes of apoptosis. Two alternative pathways for caspase activation have been well described. The extrinsic pathway results from the binding of cognate ligands to “death receptors”, whereas the intrinsic pathway involves cytochrome c release from mitochondria and formation of a multiprotein complex called the “apoptosome” [2]. Mitochondrial outer membrane permeabilization (MOMP), which leads to the release of cytochrome c and other apoptogenic factors into the cytosol, is controlled by interactions between proteins of the Bcl-2 family [3,4,5]. In both in the extrinsic and intrinsic pathways, the ubiquitin-proteasome system (UPS) plays a major role in cell death regulation by controlling the level, or the function, of many proteins of the core apoptotic machinery, notably the Bcl-2 family proteins [6,7,8,9]. 

### 1.1. The Bcl-2 Family

Bcl-2-related proteins all contain at least one of the four conserved Bcl-2 homology domains (BH1-BH4) which enable protein-protein interactions between the different members of the family. Proteins of the Bcl-2 family display either anti-apoptotic or pro-apoptotic functions. The members that inhibit apoptosis include Bcl-2, Bcl-x_L_, Mcl-1, Bfl-1/A1, Bcl-B and Bcl-w. These anti-apoptotic proteins and the pro-apoptotic effector members, such as Bax and Bak, share at least three BH domains and a similar globular structure. These two groups are thus named multi-domain proteins and they mainly reside at the mitochondria. Other pro-apoptotic members possess only the BH3 domain and display considerable sequence diversity outside of this region. These numerous “BH3-only” proteins transduce the various death stimuli to the mitochondria by binding to anti-apoptotic or pro-apoptotic multi-domain Bcl-2 family proteins. In these interactions, the amphipathic α-helical fold formed by the BH3 domain of the BH3-only proteins locates into a groove formed by the BH1, BH2 and BH3 domains of the multi-domain proteins. All BH3-only proteins can act as sensitizers by inactivating anti-apoptotic Bcl-2 family proteins, but some of them (e.g., tBid, Bim and Puma) can directly activate the pro-apoptotic effector proteins Bax and Bak [5,10]. The differential preferences of BH3-only proteins for binding to individual survival Bcl-2 proteins allows the integration of many signaling pathways [11]. These interactions primarily occur on intracellular membranes, particularly the mitochondrial outer membrane to which many family members are directed by a C-terminal hydrophobic transmembrane domain. It is the resulting balance in activity between anti- and pro-apoptotic proteins of the Bcl-2 family which determines the fate of the cell.

### 1.2. The Ubiquitin-Proteasome Machinery

Ubiquitination results from the covalent conjugation of ubiquitin to specific lysine residues in substrate proteins, under the sequential action of E1 (ubiquitin-activating enzyme), E2 (ubiquitin-conjugating enzyme) and E3 (ubiquitin protein-ligase) enzymes. This mono-ubiquitination can then be extended by ligation of further ubiquitin molecules to any of the seven lysine residues present in ubiquitin, thereby producing ubiquitin chains of various topologies [12]. These modifications can have diverse effects on the substrate, ranging from proteasome-dependent proteolysis [13] to modulation of protein function, subcellular distribution and/or protein-protein interactions [14]. The E3 ubiquitin-ligases confer a high degree of specificity to ubiquitination by recognizing the target proteins. These enzymes can be classified into two major groups defined by the presence of either a HECT (E6AP carboxyl terminus) or a RING (really interesting new gene) domain [15]. For HECT domain E3 ubiquitin-ligases, transfer of ubiquitin from the E2 to a substrate lysine involves an obligate thioester intermediate with the active-site cysteine of the C-terminal HECT domain. The N-terminal domain, which varies among the different HECT domain proteins, is involved in specific substrate recognition. The vast majority of E3 ubiquitin-ligases belong to the group of RING-containing E3s [16]. The RING is a Zn^2+^-coordinating domain defined by a pattern of conserved cysteine and histidine residues that form a “cross-brace” structure. The RING domain mediates the direct transfer of ubiquitin from the E2 to the substrate by serving as a scaffold that brings E2 and substrate together. RING finger E3 ubiquitin-ligases can function as monomers, dimers or multi-subunit complexes. Multi-subunit RING E3s are exemplified by the CRL (cullin RING ligase) superfamily [17], which includes the SCF complex, consisting of SKP1 (S-phase kinase-associated protein 1), cullin and F-box protein, and the more elaborate APC/C (anaphase-promoting complex/cyclosome). In the SCF complex, an interchangeable F-box protein confers substrate specificity, while a RING-containing subunit binds the E2. The APC/C consists of at least thirteen different subunits, including the E2-binding RING finger protein APC11 and one of the two co-activators, Cdc20 and Cdh1, which recognizes the substrate. Ubiquitination is a reversible post-translational modification that can be opposed by deubiquitinating enzymes (DUBs). These enzymes, which exhibit specificity towards the various ubiquitin chain topologies, can be grouped into five distinct families: four cysteine protease families and one metalloprotease family [18]. Since the early 1990s, ubiquitination and deubiquitination have been reported to modulate key proteins involved in apoptosis regulation [6,7,8,9,19], notably the Bcl-2 family members [7]. Among them, Mcl-1 has been the focus of extensive studies that we will attempt to summarize in this review.

## 2. Mcl-1 is a Crucial Prosurvival Protein

Mcl-1 (Myeloid cell leukemia 1) is an anti-apoptotic member of the Bcl-2 family that was originally identified as an immediate early gene expressed during TPA-mediated differentiation of a human myeloid leukemia cell line [20]. Since then, Mcl-1 has been shown to be widely expressed in many tissues [21]. Among pro-survival Bcl-2-family members, Mcl-1 is unique in that it is essential for early embryonic development and for the survival of multiple cell lineages in the adult [22]. Indeed, Mcl-1 deficiency results in peri-implantation embryonic lethality [23]. Moreover, the analysis of different conditional KO mice revealed Mcl-1-dependent survival of many cell types including lymphocytes [24,25,26], hematopoietic stem cells [27], neutrophils [28,29], neurons [30,31], hepatocytes [32], cardiomyocytes [33] and immunoglobulin-secreting plasma cells [34]. In comparison, other anti-apoptotic Bcl-2 family members are more dispensable.

**Figure 1:**
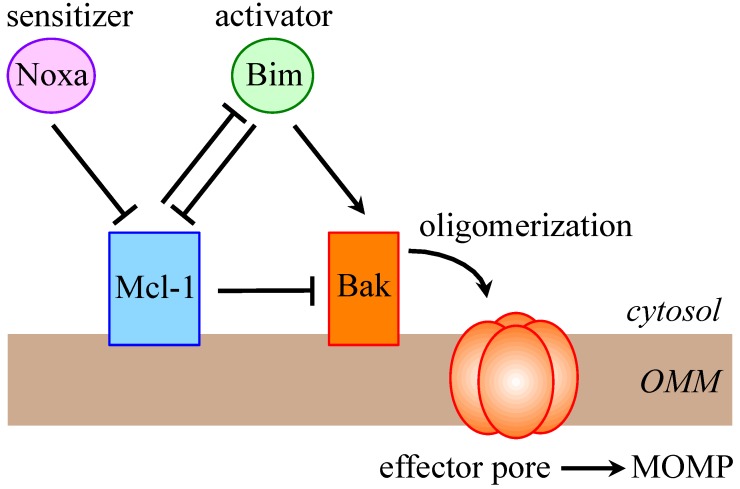
Unified model for Mcl-1 regulation of Bak-dependent mitochondrial outer membrane permeabilization (MOMP). Mcl-1 and Bak are constitutively anchored to the outer mitochondrial membrane (OMM). Mcl-1 can directly bind Bak and maintain it in an inactive conformation. Alternatively, Mcl-1 can sequester direct activator BH3-only proteins, such as Bim, and prevent them from activating Bak. Sensitizer BH3-only proteins, such as Noxa, can relieve Bak inhibition by competing with Mcl-1 for binding the BH3 domain of Bak. Activated Bak forms pore in the OMM to release cytochrome c, activate caspases and induce apoptosis. Similar mechanisms are relevant for Bax/Mcl-1 interactions.

The pro-survival role of Mcl-1 that is the best documented is the inhibition of MOMP and cytochrome c release from mitochondria. The exact mechanism underlying this effect has been extensively studied and is a part of the general controversy about Bcl-2 family protein-protein interactions. According to the unified model that is being widely accepted [4,5,35], Mcl-1 blocks MOMP by inactivating the pro-apoptotic proteins Bak and Bax. Depending on the cellular stress, Mcl‑1 may either sequester the direct-activator BH3-only proteins Bim, Puma and tBid, or directly bind the BH3 domains of Bak and Bax, or act by both mechanisms. On the other hand, Mcl-1 can be antagonized by sensitizer BH3-only proteins such as Noxa, which induce dissociation of Bak and Bax from Mcl-1 (Figure 1) [4,5].

Pro-survival functions of Mcl-1 extend beyond inhibition of cytochrome c release. Indeed, recent data suggest that Mcl-1 is also involved in autophagy regulation [36,37], by interacting with the BH3-containing autophagy inducer Beclin-1 [38,39]. Moreover, an N-terminally truncated form of Mcl-1, which is imported into the mitochondrial matrix, has been found to promote the maintenance of normal mitochondrial morphology and energy production, thereby reducing the production of deleterious reactive oxygen species [22,33,40].

Due to its crucial role in cell survival, Mcl-1 has a very high oncogenic potential. In fact, Mcl-1 is one of the most highly amplified genes across a variety of solid and haematological human malignancies [41]. In many cancers, Mcl-1 appears to be essential for cancer cells to overcome oncogenic stress-induced apoptosis. For example, Mcl-1 is critical for the development and maintenance of acute myeloid leukemia [42,43]. Furthermore, a high level of Mcl-1 is often associated with chemotherapeutic resistance and relapse [44,45]. Notably, Mcl-1 is responsible for the resistance to the new generation of BH3 mimetic cancer therapies, including ABT-737 and ABT-263, which bind to the anti-apoptotic proteins Bcl-2, Bcl-x_L _and Bcl-w to disrupt their interaction with the pro-apoptotic Bcl-2 family members [46,47,48]. Thus, Mcl-1 is an attractive and potential therapeutic target in a number of malignancies and is the focus of many studies (reviewed in [22,49]).

## 3. Multi-Level Regulation of Mcl-1

As Mcl-1 is essential for the survival of multiple cell lineages, and as Mcl-1 overexpression contributes to tumorigenesis, it is of crucial importance that its level and function are strictly controlled. Indeed, Mcl-1 is submitted to complex transcriptional, post-transcriptional and post-translational regulation processes.

### 3.1. Transcriptional, Post-Transcriptional and Translational Regulation of Mcl-1

Transcriptional regulation of Mcl-1 has been extensively studied (for reviews, see [50,51]). Multiple growth factors and cytokines, acting on a number of well-known signal transduction pathways (e.g., MAP kinases, PI3K/Akt and JAK/STAT) and downstream transcription factors, have been shown to induce Mcl-1 transcription, whereas the Mcl-1 promoter has been reported to be directly repressed by E2F1 [52]. Mcl-1 is also subject to post-transcriptional and translational regulation. For example, the mRNA of Mcl-1 can be alternatively spliced to produce two pro-apoptotic shortened forms of Mcl-1, named Mcl‑1_S_ and Mcl‑1_ES_, which do not interact with any Bcl-2 family members, except with full length Mcl-1 [53,54]. Moreover, the rate of Mcl-1 translation is tightly regulated and the Mcl-1 mRNA has a very short half-life [55]. Several miRNAs have been shown to inhibit the translation of the Mcl-1 transcript. One well-documented example is miR29 which reduces Mcl-1 protein expression by directly binding the Mcl-1 3'UTR [56]. Moreover, a screen for miRNAs that sensitize cells to ABT-263 identified 12 miRNAs that reduce Mcl-1 protein expression [57]. The RNA binding protein CUGBP2 can also bind to Mcl-1 mRNA 3'UTR and inhibits its translation [58]. In addition, eIF2α and mTORC1 have been found to modulate Mcl-1 translation, thereby coupling global control of mRNA translation to apoptosis regulation [59,60]. In these different examples, increased expression of full-length Mcl-1 resulted in improved cell survival, whereas inhibition of its expression was systematically associated with cell death induction or sensitization to apoptosis.

### 3.2. Post-Translational Regulation of Mcl-1

**Figure 2:**
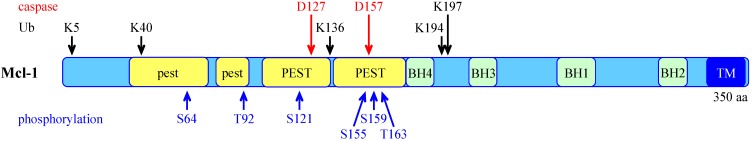
Schematic representation of the human Mcl-1 protein showing the functional regions and post-translational modification sites. These include the transmembrane domain (TM), Bcl-2 homology domains (BH1-4), weak (lower case) and strong (upper case) PEST sequences, sites of ubiquitination (Ub), caspase cleavage and phosphorylation sites.

Unlike other Bcl-2-related survival proteins, Mcl-1 harbors a long unstructured N-terminus that appears to be involved in different post-translational modifications of Mcl-1 (Figure 2) [51]. For example, it has been shown that, during apoptosis, Mcl-1 protein can be cleaved by caspases and granzyme B, at two distinct sites (Asp127 and Asp157) within the N-terminus [61,62,63,64,65]. Some studies reported that cleavage at these sites impairs the anti-apoptotic properties of Mcl-1 [61,63], or even converts it into a pro-apoptotic protein [62,65]. Cleavage of Mcl-1 thus appears to be a process through which apoptotic cells can inactivate residual Mcl-1 that could act as a brake to the achievement of cell death. The N-terminus of Mcl-1 contains PEST regions [20], enriched in proline (P), glutamate (E), serine (S) and threonine (T) residues, which are common features of rapidly degraded proteins [66]. These regions contain the two caspase cleavage sites of Mcl-1 and many phosphorylation sites (Figure 2). Differential phosphorylation of Mcl-1 at specific sites has been reported to result in different outcomes. For example, the cell cycle-dependent phosphorylation of Ser64 by CDK1, CDK2 and JNK1 enhances the anti-apoptotic function of Mcl-1 by increasing its interaction with pro-apoptotic members of the Bcl-2 family, without modifying its half-life [67]. Two independent groups have also shown that Mcl-1 phosphorylation at Thr92 and Thr163, stimulated by TPA-induced ERK activation, stabilizes Mcl-1 in some cancer cell lines [68,69,70]. Likewise, Ser121 and Thr163 have been found to be phosphorylated by JNK [71,72]. In hepatocytes, this JNK-mediated phosphorylation stabilizes Mcl-1 and affords protection against TNF-induced apoptosis [72], whereas in endothelial cells submitted to oxidative stress, this phosphorylation reduces the anti-apoptotic effect of Mcl-1 [71]. Moreover, phosphorylation of Mcl-1 at Ser155, Ser159 and Thr163, in a different cellular context, has been shown to favour Mcl-1 degradation by the UPS, as discussed below. 

## 4. Control of Mcl-1 Protein Level by the Ubiquitin-Proteasome System

One of the most prominent features of Mcl-1, that sets it apart from the other anti-apoptotic proteins of the Bcl-2 family, is its short half-life. The rapid degradation of Mcl-1 was first attributed to the proteasome in 2003 by two independent groups, in HeLa cells undergoing apoptosis following UV irradiation [73] and adenovirus infection [74]. This was later confirmed by other groups in different systems [75,76,77]. It appeared at that time that the elimination of Mcl-1 was required for the initiation of apoptosis. Indeed, proteasome inhibitors could block the elimination of Mcl-1 and prolong survival of Mcl-1 expressing cells, whereas they were unable to prevent apoptosis after Mcl-1 knock-down [73]. Then, the poly-ubiquitination of Mcl-1 was demonstrated and five lysine residues involved in Mcl-1 ubiquitination were identified [78] (Figure 2). In the following years, a number of studies described how these mechanisms could be regulated by different enzymes.

### 4.1. The E3 Ubiquitin-Ligases of Mcl-1

To date, five E3 ubiquitin-ligases have been involved in the ubiquitination of Mcl-1, targeting it for proteasomal degradation, four of which were demonstrated to directly ubiquitinate Mcl-1.

#### 4.1.1. Mule

The first E3 ubiquitin-ligase of Mcl-1 was identified in 2005, by a biochemical fractionation strategy using *in vitro* ubiquitination of Mcl-1 as a read-out [78]. It was named Mule for “Mcl-1 ubiquitin ligase E3” but was originally known as LASU1 and ARF-BP1. Mule was identified in parallel in a genome wide search for new BH3-containing proteins [79], as it contains a well-conserved BH3 domain. It is of note that the BH3 domain of Mule interacts with Mcl-1 but not with Bcl-2, Bcl-x_L_ or Bax [78,79]. Mule is a 480 kDa protein that belongs to the HECT domain family of E3 ubiquitin-ligases. Mule has been demonstrated to ubiquitinate Mcl-1 *in vitro* and to be necessary for Mcl-1 poly-ubiquitination in HeLa cells [78,79]. Indeed, inhibition of Mule expression by RNA interference stabilized Mcl-1 protein, resulting in an attenuation of DNA damage-induced apoptosis [78].

**Figure 3:**
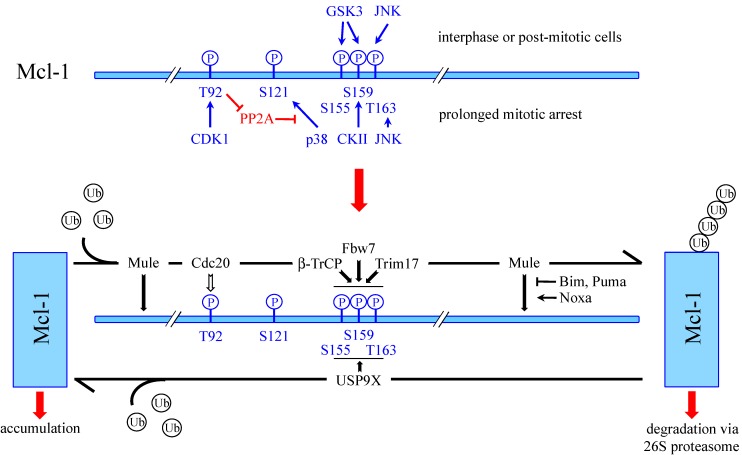
Regulation of Mcl-1 stability. Mcl-1 can be phosphorylated by several protein kinases (in blue) at indicated residues. In interphase or post-mitotic cells, JNK phosphorylates Thr163 which primes Mcl-1 for phosphorylation by GSK3 at Ser159 and Ser155. During prolonged mitotic arrest, p38, CKII and JNK phosphorylate Ser121, Ser159 and Thr163. CDK1 may indirectly enhance phosphorylation at these sites by phosphorylating Thr92, thereby triggering the dissociation of the phosphatase PP2A that would otherwise dephosphorylate Mcl-1. Phosphorylation of Ser155, Ser159 and Thr163 drives the recognition of Mcl-1 by its E3 ubiquitin-ligases SCF^β-TrCP^, SCF^Fbw7^ and Trim17 (in black). In addition, phosphorylation at Thr92 is required for Cdc20-mediated ubiquitination of Mcl-1, although direct ubiquitination of Mcl-1 by APC/C^Cdc20^ has not been demonstrated. In addition, the E3 ubiquitin-ligase Mule can interact either with the C-terminus of Mcl-1 through its BH3 domain, or within the N-terminal 30 amino acids of Mcl-1. Binding of Mule does not depend on Mcl-1 phosphorylation but it can be inhibited by the BH3-only proteins Bim and Puma. In contrast, Noxa increases the association between Mule and Mcl-1 through an as yet unknown mechanism. Ubiquitination of Mcl-1 targets it for proteasomal degradation. It can be opposed by the deubiquitinase USP9X that directly removes degradative Lys-48-linked polyubiquitin chains from Mcl-1, which results in Mcl‑1 stabilization. Phosphorylation at Ser155, Ser159 and Thr163 inhibits the binding of USP9X to Mcl-1. In cancer cells in which Mcl-1 degradation is not dependent on GSK3, ERK-mediated phosphorylation of Thr163 stabilizes Mcl-1 (not depicted here).

Mule requires its BH3 domain to target Mcl-1 for rapid degradation [78,79]. However, the Mule BH3 peptide has a very low binding affinity for Mcl-1 compared to BH3 peptides from other Mcl-1 binding partners (Bak, Bim, Bid, Puma and Noxa) [80]. These BH3 peptides should thus easily displace Mule from Mcl-1. Indeed, overexpression of Bim [81] or Puma [82] has been shown to stabilize Mcl-1 by preventing its interaction with Mule [79] (Figure 3), whereas knock-down of Bim increased Mcl-1 degradation [83]. Conversely, binding of Noxa to Mcl-1 was found to trigger the proteasomal degradation of Mcl-1 [77,81], by favoring its interaction with Mule [84] (Figure 3). The differential effects of the two BH3-only proteins Bim and Noxa seem to rely on a discrete C-terminal sequence of the Noxa BH3 domain [81]. Formation of the Noxa/Mcl-1 complex might promote the binding of Mule through a distinct mechanism than docking of the Mule BH3 into the BH3-binding groove of Mcl-1. Indeed, a second site of interaction with Mule exists within the N-terminal 30 amino acids of Mcl-1 [80] (Figure 3). This should permit Mule to bind Mcl-1 even when a BH3-only protein interacts with Mcl-1.

In cultured cell lines, Mule seems to be responsible for constitutive Mcl-1 degradation [78,79]. However, target gene deletion in the mouse, suggests that Mule-dependent ubiquitination of Mcl-1 is induced only by specific stimuli. Indeed, Mule attracted considerable interest because it targets many substrates such as p53, c-Myc, cdc6 and N-Myc, in addition to Mcl-1 (reviewed in [85]). Therefore, several conditional knockout mice have been generated. It appears that basal Mcl‑1 protein level is largely unaffected in Mule-deficient cells [85,86]. In contrast, etoposide-induced degradation of Mcl-1 and apoptosis are efficiently blocked by Mule deficiency [86]. This suggests that Mcl-1 degradation may involve other E3 ubiquitin-ligases or ubiquitin-independent processes, depending on the conditions.

#### 4.1.2. Phosphorylation-Dependent Degradation of Mcl-1

Several independent studies have shown that phosphorylation of Mcl-1 by GSK3 (Glycogen Synthase Kinase 3) at Ser155, and/or Ser159 leads to significant decrease in Mcl-1 protein level [87,88,89,90,91,92]. In this context, it was demonstrated that prior phosphorylation of Mcl-1 by JNK at Thr163 (Thr144 in mouse) is required for the subsequent phosphorylation of Mcl-1 by GSK3 [90] (Figure 3). Indeed, priming phosphorylation of the GSK3 consensus site is often required for the docking of the protein kinase to its substrates [93]. Phosphorylation of Mcl-1 by the coordinated activity of JNK and GSK3 appears to create a phosphodegron mediating the proteasomal degradation of Mcl-1. Indeed, phosphorylation-defective mutations of these different serine and threonine residues stabilized Mcl-1 and increased protection from apoptosis following growth factor withdrawal [89,92], expression of constitutively active GSK3 [87], UV irradiation [90] and anti-cancer drugs [87,88]. In some cases, Mcl-1 ubiquitination was also shown to be reduced by phosphorylation-defective mutations suggesting that Mcl-1 degradation promoted by phosphorylation is ubiquitin-dependent [87,88,89,92]. Although the studies mentioned above all showed that Thr163 phosphorylation promotes Mcl-1 degradation [87,88,89,90,91,92], others reported that phosphorylation by ERK at the same site stabilizes Mcl-1 [48,68,69,70]. A possible explanation for this apparent discrepancy has been recently proposed [70]. Indeed, studies showing a stabilization effect have been performed in various cancer cell lines in which Mcl-1 degradation was found to be independent of the GSK3 pathway [70]. This is consistent with emerging findings showing that Mcl-1 degradation through this pathway is impaired in many different types of cancer [69,94]. Overall, Thr163 phosphorylation can prime GSK3-targeted Mcl-1 degradation to promote death in normal cells, whereas in cancer cells in which degradation is not dependent on this pathway, ERK-mediated phosphorylation of Thr163 is associated with Mcl-1 stabilization and drug resistance.

#### 4.1.3. SCF^β-TrCP^

The first E3 ubiquitin-ligase accounting for GSK3-dependent proteasomal degradation of Mcl-1 was identified as SCF^β-TrCP ^(beta-transducin repeats-containing protein) [87]. Indeed, it was demonstrated that Mcl-1 phosphorylation at Ser155, Ser159 and Thr163 by GSK3 facilitates its association with the F-box protein β-TrCP [87] (Figure 3). Moreover knock-down of β-TrCP increased Mcl‑1 levels, whereas overexpression of β-TrCP induced Mcl-1 ubiquitination in a phosphorylation- and F-box-dependent manner. In addition, the SCF^β-TrCP^ complex was able to ubiquitinate Mcl-1 *in vitro* [87]. The role of this SCF E3 ubiquitin-ligase in the GSK3-dependent ubiquitination and degradation of Mcl-1 has recently been confirmed in lung cancer cell lines undergoing apoptosis following Akt inhibition [95]. Interestingly, SCF^β-TrCP^ has also been reported to target Bim_EL_ for degradation in a phosphorylation-dependent manner [96]. Apart from these two Bcl-2 family members, SCF^β-TrCP^ ubiquitinates several substrates that are involved in cell division regulation and various transduction pathways, which, in turn, are essential for many aspects of tumorigenesis [97].

#### 4.1.4. SCF^Fbw7^

Another SCF E3 ubiquitin-ligase, containing the F-box protein Fbw7 (F-box and WD repeat domain-containing 7) as a substrate-recognition component, has also been implicated in Mcl-1 ubiquitination [88,98]. Fbw7 is a well-characterized tumor suppressor which is frequently lost in diverse types of cancers. The SCF^Fbw7^ complex targets numerous oncoproteins including c-Myc, cyclin E, Notch and c-Jun for ubiquitination and degradation [99]. Two independent studies additionally showed that SCF^Fbw7^ mediates Mcl-1 ubiquitination in a phosphorylation-dependent manner [88,98] (Figure 3). Indeed, loss of Fbw7 in both human and mouse resulted in accumulation of Mcl-1, due to increased half-life, providing resistance to different chemotherapeutics [88,98]. In contrast, reintroduction of wild type Fbw7 dramatically reduced Mcl-1 levels and decreased its stability [88,98]. Moreover, recombinant Mcl-1 was ubiquitinated *in vitro* by the reconstituted Fbw7-containing SCF complex [98]. In both studies, mutation of the phosphorylation sites Ser159 and Thr163 impaired the interaction between Mcl-1 and Fbw7 and reduced Mcl-1 degradation [88,98]. However, the protein kinases directing Mcl-1 recruitment to Fbw7 differed from one study to another. In asynchronous cells, pharmacological inhibition of GSK3 decreased the binding of Fbw7 to Mcl-1, and Fbw7 promoted Mcl-1 ubiquitination when it was co-transfected with GSK3 [88] (Figure 3). However, during mitotic arrest, activities of JNK, p38, CKII and CDKI, but not GSK3, were found to regulate Mcl-1 degradation, by phosphorylating Mcl-1 and promoting Fbw7 binding [98,100]. In these conditions, JNK, p38 and CKII directly phosphorylated Mcl-1, while CDK1 indirectly enhanced phosphorylation of Mcl-1 at Ser121, Ser159 and Thr63, by phosphorylating Thr92, and thereby driving the dissociation of the phosphatase PP2A from Mcl-1 [98,100] (Figure 3). Therefore, the kinases involved in the targeting of Mcl-1 by Fbw7 seem to differ depending on the cellular context.

#### 4.1.5. APC/CC^dc20^

During prolonged mitotic arrest, the multi-subunit RING E3 ubiquitin-ligase APC/C^Cdc20^ has also been involved in Mcl-1 degradation [101], in addition to SCF^Fbw7^. Indeed, in cells arrested in mitosis by microtubule poisons, Mcl-1 proteasomal degradation was found to depend on the substrate-recognition co-activator Cdc20, and to require prior phosphorylation of Mcl-1 at Thr92 by CDK1/cyclin B1 [101] (Figure 3). However this phosphorylation did not modify the interaction between Mcl-1 and Cdc20, and there is no firm evidence as yet that APC/C^Cdc20^ directly mediates Mcl-1 ubiquitination. Nevertheless, stabilization of Mcl-1 by mutation of Thr92 inhibited apoptosis induced by prolonged mitotic arrest [101]. Therefore, control of Mcl-1 instability by APC/C^Cdc20 ^constitutes a direct link between the regulation of mitosis and the temporal control of apoptosis [100]. In a normal mitosis, the level of Mcl-1 steadily decreases but the reduction in Mcl-1 is insufficient to trigger apoptosis, whereas during prolonged mitotic arrest, the level of Mcl-1 eventually drops below a protective threshold and apoptosis is initiated.

#### 4.1.6. Trim17

The latest E3 ubiquitin-ligase of Mcl-1 that has been formerly identified is Trim17 (Tripartite motif containing 17) [92]. Trim17 is a member of the TRIM family that constitutes one of the largest classes of single-protein RING-containing E3 ubiquitin-ligases [102,103]. Trim17, also known as terf (testis RING finger protein) was first isolated from rat and human testis cDNA libraries [104], but it is also expressed in spleen, thymus and to a lesser extent in liver, kidney and brain [105]. Little is known about the cellular function of Trim17. It has been implicated in the regulation of cell proliferation, possibly by promoting the degradation of the kinetochore protein ZWINT [106]. In addition, Trim17 expression has been demonstrated to be both necessary and sufficient for neuronal apoptosis [107]. This pro-apoptotic effect appears to depend on the E3 ubiquitin-ligase activity of Trim17 and on the Bax-dependent mitochondrial pathway [107]. In apoptotic neurons deprived of survival factors, as described in other cell types [87,88,89,90,91], ubiquitination and degradation of Mcl-1 depend on its prior phosphorylation by GSK3 [92]. Knock-down of Trim17 expression increased the protein level and half-life of Mcl-1, and reduced its ubiquitination level in neurons. In contrast, overexpression of Trim17 decreased the protein level of Mcl-1 in a proteasome-dependent manner. This effect was abolished by inhibition of GSK3 and JNK, and by phosphorylation-defective mutations of Ser140 and Thr144 of mouse Mcl-1 (corresponding to Ser159 and Thr163 in human Mcl-1) [92]. Moreover Trim17 could ubiquitinate recombinant Mcl-1 *in vitro* [92]. Impairment of Mcl-1 phosphorylation, either by kinase inhibition or point mutations, not only decreased Mcl-1 ubiquitination and degradation, but also disrupted the physical interaction between Trim17 and Mcl-1, the resulting stabilization of Mcl-1 increasing its neuroprotective effect [92]. Therefore, Trim17 appears to be a physiological E3 ubiquitin-ligase of Mcl-1 in neurons that requires phosphorylation at Ser159 and Thr163 to bind Mcl-1 (Figure 3). Nonetheless, Trim17 expression is not restricted to neurons, and it is possible that it mediates Mcl-1 ubiquitination in other cell types.

### 4.2. Deubiquitination of Mcl-1 by USP9X

Ubiquitination is a reversible event that can be counteracted by specific enzymes. A deubiquitinase from the USP family, USP9X (ubiquitin specific peptidase 9 X-linked), was identified among proteins co-immunoprecipitating with Mcl-1 [108]. It has been shown to remove poly-ubiquitin chains from Mcl-1, thereby stabilizing it and leading to apoptosis resistance. Indeed, knock-down of USP9X reduced the half-life of Mcl-1 and increased its conjugation to Lys48-linked poly-ubiquitin chains [108] that generally target proteins for proteasomal degradation. USP9X also deubiquitinated Mcl-1 *in vitro*, and generated free mono-ubiquitin [108]. Therefore, USP9X appears to stabilize Mcl-1 by removing its degradative Lys48-linked poly-ubiquitin chains. Direct binding of USP9X was essential for Mcl-1 stabilization. Interestingly, phosphorylation-defective mutations of the Mcl‑1 residues Ser155, Ser159 and Thr163 to alanines enhanced the interaction between Mcl-1 and USP9X. In contrast, phosphomimetic mutations of the three residues to aspartic acids decreased the interaction [108]. Moreover, inhibition of PI3K, in order to activate GSK3, also disrupted the binding of USP9X to Mcl-1, whereas GSK3 inhibition prevented UV-induced dissociation of USP9X from Mcl-1 [108]. Therefore, phosphorylation at Ser155, Ser159 and Thr163 not only drives the binding of E3 ubiquitin-ligases such as SCF^β-TrCP^, SCF^Fbw7^ or Trim17, but also disrupts the binding of the deubiquitinase USP9X (Figure 3). It is thus possible that E3 ubiquitin-ligases and USP9X compete for Mcl-1 binding at this crucial phosphodegron.

### 4.3. Ubiquitin-Independent Degradation of Mcl-1

The involvement of ubiquitination in Mcl-1 proteasomal degradation has been questioned in a study using a Mcl-1 mutant in which all 14 lysine residues were replaced by arginines (Mcl-1^KR^) [109]. This mutant protein could not be ubiquitinated. However, it was eliminated by the proteasome at a rate similar to wild type Mcl-1, when stably expressed in *Mcl-1*-deleted MEFs. Importantly, the half-lives of wild type Mcl-1 and Mcl-1^KR^ were similar under basal conditions, following UV irradiation and even when a constitutively active form of Akt was co-expressed [109]. Moreover, the degradation of wild-type Mcl-1 was not affected when the E1 activity was blocked, whereas the half-life of p53 was strongly increased in these conditions [109]. To further assess the role of ubiquitination in Mcl-1 degradation *in vivo*, transgenic mice expressing epitope-tagged versions of wild-type Mcl-1 or Mcl-1^KR^ were generated. In thymocytes and T lymphocytes derived from these mice, both forms of Mcl-1 were degraded at a similar rate [109]. Lastly, unmodified, *in vitro*-translated Mcl‑1 could be degraded in a cell-free system by the 20S proteasome in the absence of ubiquitination [109]. This work thus indicates that Mcl-1 can be directly targeted by the proteasome, in an ubiquitin-independent manner, as it has been reported for other proteins [110,111].

However, these observations do not exclude the possibility that ubiquitination can accelerate Mcl-1 elimination in response to certain apoptotic stimuli. Indeed, overexpression of Mule enhanced degradation of wild type Mcl-1 whereas it did not influence the turn-over of Mcl-1^KR^ [109]. In addition, mutagenesis of critical lysine residues has been found by others to decrease ubiquitination and extend the half-life of Mcl-1 in different cell types [78,88,92]. This supports the notion that ubiquitination of Mcl-1 is required for its proteasomal degradation, at least in some conditions. In some studies, the proteasomal degradation of Mcl-1 appeared to be constitutive, as its half-life was the same in the presence or the absence of the death stimuli [73]. However, many others studies reported that the degradation of Mcl-1 was accelerated during apoptosis [74,88,98], notably in growth-factor dependent cells [75,87,89,91,92], suggesting that the degradation of Mcl-1 can be accelerated by pro-apoptotic stimuli. Moreover the half-life of Mcl-1 was shown to be modulated by silencing of the Mule, β-TrCP, Fbw7 and Trim17 E3 ubiquitin-ligases, as well as the USP9X deubiquitinase [78,87,88,92,98,108]. Overall, it is possible that Mcl-1 is normally degraded in an ubiquitin-dependent manner, notably in response to cellular stresses. However, if Mcl-1 ubiquitination is blocked, its degradation may still occur normally by an alternative ubiquitin-independent mechanism.

## 5. Conclusions

Accumulating evidence indicates that Mcl-1 is a critical pro-survival protein for a myriad of cell types, under both physiological and malignant conditions. Therefore, it is not surprising that Mcl-1 protein level is strictly controlled in normal cells, and that cancer cells have a vast arsenal to ensure high levels of Mcl-1 and thereby escape apoptosis. Mcl-1 expression is regulated at multiple levels. The proteosomal degradation provides the protein with the unique ability to immediately respond to environmental signals and switch cell fate from survival to apoptosis. Highlighting the importance of this process, a plethora of pathways and enzymes have been found to modulate the ubiquitination and degradation of Mcl-1. Over the past few years, five E3 ubiquitin-ligases, one deubiquitinase, more than six protein kinases and ubiquitin-independent proteasomal degradation have been implicated in the regulation of Mcl-1 stability. The relative contributions of these different mechanisms are still unclear. The protein kinases involved in the recognition of Mcl-1 by some E3 ubiquitin ligases seem to depend on apoptotic stimuli [88,98]. Likewise, the E3 ubiquitin-ligases involved in Mcl-1 ubiquitination seem to vary depending on specific cell types and death signals. For example, Mule activity is not implicated in GSK3-dependent ubiquitination of Mcl-1 [78,87], or in Mcl-1 degradation during mitotic arrest [98,101]. Moreover, β-TrCP does not appear to play a role in Mcl-1 degradation, in the models in which Fbw7 was identified as a Mcl-1 E3 ubiquitin-ligase [88,98]. Therefore, different sets of kinases and E3 ubiquitin-ligases appear to control Mcl-1 levels, allowing different cell types to switch to either survival or apoptotic mode in response to various stresses. Future studies delineating the relative contributions of these different players in regulating Mcl-1 protein levels in specific conditions may help us develop more effective therapeutic strategies for the treatment of certain malignancies.

## References

[1] Fuchs Y., Steller H. (2011). Programed cell death in animal development and disease. Cell.

[2] Danial N.N., Korsmeyer S.J. (2004). Cell death: critical control points. Cell.

[3] Desagher S., Martinou J.-C. (2000). Mitochondria as the central control point of apoptosis. Trends Cell Biol..

[4] Czabotar P.E., Lessene G., Strasser A., Adams J.M. (2014). Control of apoptosis by the BCL-2 protein family: implications for physiology and therapy. Nat. Rev. Mol. Cell. Biol..

[5] Moldoveanu T., Follis A.V., Kriwacki R.W., Green D.R. (2014). Many players in BCL-2 family affairs. Trends Biochem. Sci..

[6] Broemer M., Meier P. (2009). Ubiquitin-mediated regulation of apoptosis. Trends Cell Biol..

[7] Neutzner A., Li S., Xu S., Karbowski M. (2012). The ubiquitin/proteasome system-dependent control of mitochondrial steps in apoptosis. Semin. Cell Dev. Biol..

[8] Thompson S.J., Loftus L.T., Ashley M.D., Meller R. (2008). Ubiquitin-proteasome system as a modulator of cell fate. Curr. Opin. Pharmacol..

[9] Vucic D., Dixit V.M., Wertz I.E. (2011). Ubiquitylation in apoptosis: a post-translational modification at the edge of life and death. Nat. Rev. Mol. Cell. Biol..

[10] Happo L., Strasser A., Cory S. (2012). BH3-only proteins in apoptosis at a glance. J. Cell Sci..

[11] Chen L., Willis S.N., Wei A., Smith B.J., Fletcher J.I., Hinds M.G., Colman P.M., Day C.L., Adams J.M., Huang D.C. (2005). Differential targeting of prosurvival Bcl-2 proteins by their BH3-only ligands allows complementary apoptotic function. Mol. Cell.

[12] Komander D., Rape M. (2012). The ubiquitin code. Annu. Rev. Biochem..

[13] Glickman M.H., Ciechanover A. (2002). The ubiquitin-proteasome proteolytic pathway: destruction for the sake of construction. Physiol. Rev..

[14] Chen Z.J., Sun L.J. (2009). Nonproteolytic functions of ubiquitin in cell signaling. Mol. Cell.

[15] Metzger M.B., Hristova V.A., Weissman A.M. (2012). HECT and RING finger families of E3 ubiquitin ligases at a glance. J. Cell Sci..

[16] Deshaies R.J., Joazeiro C.A. (2009). RING domain E3 ubiquitin ligases. Annu. Rev. Biochem..

[17] Petroski M.D., Deshaies R.J. (2005). Function and regulation of cullin-RING ubiquitin ligases. Nat. Rev. Mol. Cell. Biol..

[18] Clague M.J., Coulson J.M., Urbe S. (2012). Cellular functions of the DUBs. J. Cell Sci..

[19] Ramakrishna S., Suresh B., Baek K.H. (2011). The role of deubiquitinating enzymes in apoptosis. Cell. Mol. Life Sci..

[20] Kozopas K.M., Yang T., Buchan H.L., Zhou P., Craig R.W. (1993). MCL1, a gene expressed in programed myeloid cell differentiation, has sequence similarity to BCL2. Proc. Natl. Acad. Sci. U.S.A..

[21] Krajewski S., Bodrug S., Krajewska M., Shabaik A., Gascoyne R., Berean K., Reed J.C. (1995). Immunohistochemical analysis of Mcl-1 protein in human tissues. Differential regulation of Mcl-1 and Bcl-2 protein production suggests a unique role for Mcl-1 in control of programed cell death *in vivo*. Am. J. Pathol..

[22] Perciavalle R.M., Opferman J.T. (2013). Delving deeper: MCL-1's contributions to normal and cancer biology. Trends Cell Biol..

[23] Rinkenberger J.L., Horning S., Klocke B., Roth K., Korsmeyer S.J. (2000). Mcl-1 deficiency results in peri-implantation embryonic lethality. Genes Dev..

[24] Opferman J.T., Letai A., Beard C., Sorcinelli M.D., Ong C.C., Korsmeyer S.J. (2003). Development and maintenance of B and T lymphocytes requires antiapoptotic MCL-1. Nature.

[25] Vikstrom I., Carotta S., Luthje K., Peperzak V., Jost P.J., Glaser S., Busslinger M., Bouillet P., Strasser A., Nutt S.L. (2010). Mcl-1 is essential for germinal center formation and B cell memory. Science.

[26] Dzhagalov I., Dunkle A., He Y.W. (2008). The anti-apoptotic Bcl-2 family member Mcl-1 promotes T lymphocyte survival at multiple stages. J. Immunol..

[27] Opferman J.T., Iwasaki H., Ong C.C., Suh H., Mizuno S., Akashi K., Korsmeyer S.J. (2005). Obligate role of anti-apoptotic MCL-1 in the survival of hematopoietic stem cells. Science.

[28] Dzhagalov I., St John A., He Y.W. (2007). The antiapoptotic protein Mcl-1 is essential for the survival of neutrophils but not macrophages. Blood.

[29] Steimer D.A., Boyd K., Takeuchi O., Fisher J.K., Zambetti G.P., Opferman J.T. (2009). Selective roles for antiapoptotic MCL-1 during granulocyte development and macrophage effector function. Blood.

[30] Arbour N., Vanderluit J.L., Le Grand J.N., Jahani-Asl A., Ruzhynsky V.A., Cheung E.C., Kelly M.A., MacKenzie A.E., Park D.S., Opferman J.T. (2008). Mcl-1 is a key regulator of apoptosis during CNS development and after DNA damage. J. Neurosci..

[31] Malone C.D., Hasan S.M., Roome R.B., Xiong J., Furlong M., Opferman J.T., Vanderluit J.L. (2012). Mcl-1 regulates the survival of adult neural precursor cells. Mol. Cell. Neurosci..

[32] Vick B., Weber A., Urbanik T., Maass T., Teufel A., Krammer P.H., Opferman J.T., Schuchmann M., Galle P.R., Schulze-Bergkamen H. (2009). Knockout of myeloid cell leukemia-1 induces liver damage and increases apoptosis susceptibility of murine hepatocytes. Hepatology.

[33] Wang X., Bathina M., Lynch J., Koss B., Calabrese C., Frase S., Schuetz J.D., Rehg J.E., Opferman J.T. (2013). Deletion of MCL-1 causes lethal cardiac failure and mitochondrial dysfunction. Genes Dev..

[34] Peperzak V., Vikstrom I., Walker J., Glaser S.P., LePage M., Coquery C.M., Erickson L.D., Fairfax K., Mackay F., Strasser A. (2013). Mcl-1 is essential for the survival of plasma cells. Nat. Immunol..

[35] Llambi F., Moldoveanu T., Tait S.W., Bouchier-Hayes L., Temirov J., McCormick L.L., Dillon C.P., Green D.R. (2011). A unified model of mammalian BCL-2 protein family interactions at the mitochondria. Mol. Cell.

[36] Germain M., Nguyen A.P., Le Grand J.N., Arbour N., Vanderluit J.L., Park D.S., Opferman J.T., Slack R.S. (2011). MCL-1 is a stress sensor that regulates autophagy in a developmentally regulated manner. EMBO J..

[37] Thomas R.L., Roberts D.J., Kubli D.A., Lee Y., Quinsay M.N., Owens J.B., Fischer K.M., Sussman M.A., Miyamoto S., Gustafsson A.B. (2013). Loss of MCL-1 leads to impaired autophagy and rapid development of heart failure. Genes Dev..

[38] Sharma A., Singh K., Mazumder S., Hill B.T., Kalaycio M., Almasan A. (2013). BECN1 and BIM interactions with MCL-1 determine fludarabine resistance in leukemic B cells. Cell Death Dis..

[39] Bonapace L., Bornhauser B.C., Schmitz M., Cario G., Ziegler U., Niggli F.K., Schafer B.W., Schrappe M., Stanulla M., Bourquin J.P. (2010). Induction of autophagy-dependent necroptosis is required for childhood acute lymphoblastic leukemia cells to overcome glucocorticoid resistance. J Clin. Invest..

[40] Perciavalle R.M., Stewart D.P., Koss B., Lynch J., Milasta S., Bathina M., Temirov J., Cleland M.M., Pelletier S., Schuetz J.D. (2012). Anti-apoptotic MCL-1 localizes to the mitochondrial matrix and couples mitochondrial fusion to respiration. Nat. Cell Biol..

[41] Beroukhim R., Mermel C.H., Porter D., Wei G., Raychaudhuri S., Donovan J., Barretina J., Boehm J.S., Dobson J., Urashima M. (2010). The landscape of somatic copy-number alteration across human cancers. Nature.

[42] Glaser S.P., Lee E.F., Trounson E., Bouillet P., Wei A., Fairlie W.D., Izon D.J., Zuber J., Rappaport A.R., Herold M.J. (2012). Anti-apoptotic Mcl-1 is essential for the development and sustained growth of acute myeloid leukemia. Genes Dev..

[43] Xiang Z., Luo H., Payton J.E., Cain J., Ley T.J., Opferman J.T., Tomasson M.H. (2010). Mcl1 haploinsufficiency protects mice from Myc-induced acute myeloid leukemia. J. Clin. Invest..

[44] Wei G., Twomey D., Lamb J., Schlis K., Agarwal J., Stam R.W., Opferman J.T., Sallan S.E., den Boer M.L., Pieters R. (2006). Gene expression-based chemical genomics identifies rapamycin as a modulator of MCL1 and glucocorticoid resistance. Cancer Cell.

[45] Wuilleme-Toumi S., Robillard N., Gomez P., Moreau P., Le Gouill S., Avet-Loiseau H., Harousseau J.L., Amiot M., Bataille R. (2005). Mcl-1 is overexpressed in multiple myeloma and associated with relapse and shorter survival. Leukemia.

[46] Konopleva M., Contractor R., Tsao T., Samudio I., Ruvolo P.P., Kitada S., Deng X., Zhai D., Shi Y.X., Sneed T. (2006). Mechanisms of apoptosis sensitivity and resistance to the BH3 mimetic ABT-737 in acute myeloid leukemia. Cancer Cell.

[47] van Delft M.F., Wei A.H., Mason K.D., Vandenberg C.J., Chen L., Czabotar P.E., Willis S.N., Scott C.L., Day C.L., Cory S. (2006). The BH3 mimetic ABT-737 targets selective Bcl-2 proteins and efficiently induces apoptosis via Bak/Bax if Mcl-1 is neutralized. Cancer Cell.

[48] Mazumder S., Choudhary G.S., Al-Harbi S., Almasan A. (2012). Mcl-1 Phosphorylation defines ABT-737 resistance that can be overcome by increased NOXA expression in leukemic B cells. Cancer Res..

[49] Ertel F., Nguyen M., Roulston A., Shore G.C. (2013). Programming cancer cells for high expression levels of Mcl1. EMBO Rep..

[50] Le Gouill S., Podar K., Harousseau J.L., Anderson K.C. (2004). Mcl-1 regulation and its role in multiple myeloma. Cell Cycle.

[51] Thomas L.W., Lam C., Edwards S.W. (2010). Mcl-1; the molecular regulation of protein function. FEBS Lett..

[52] Croxton R., Ma Y., Song L., Haura E.B., Cress W.D. (2002). Direct repression of the Mcl-1 promoter by E2F1. Oncogene.

[53] Bingle C.D., Craig R.W., Swales B.M., Singleton V., Zhou P., Whyte M.K. (2000). Exon skipping in Mcl-1 results in a bcl-2 homology domain 3 only gene product that promotes cell death. J. Biol. Chem..

[54] Kim J.H., Bae J. (2013). MCL-1ES induces MCL-1L-dependent BAX- and BAK-independent mitochondrial apoptosis. PLoS One.

[55] Yang T., Buchan H.L., Townsend K.J., Craig R.W. (1996). MCL-1, a member of the BLC-2 family, is induced rapidly in response to signals for cell differentiation or death, but not to signals for cell proliferation. J. Cell Physiol..

[56] Mott J.L., Kobayashi S., Bronk S.F., Gores G.J. (2007). mir-29 regulates Mcl-1 protein expression and apoptosis. Oncogene.

[57] Lam L.T., Lu X., Zhang H., Lesniewski R., Rosenberg S., Semizarov D. (2010). A microRNA screen to identify modulators of sensitivity to BCL2 inhibitor ABT-263 (navitoclax). Mol. Cancer Ther..

[58] Subramaniam D., Natarajan G., Ramalingam S., Ramachandran I., May R., Queimado L., Houchen C.W., Anant S. (2008). Translation inhibition during cell cycle arrest and apoptosis: Mcl-1 is a novel target for RNA binding protein CUGBP2. Am. J. Physiol. Gastrointest. Liver Physiol..

[59] Mills J.R., Hippo Y., Robert F., Chen S.M., Malina A., Lin C.J., Trojahn U., Wendel H.G., Charest A., Bronson R.T. (2008). mTORC1 promotes survival through translational control of Mcl-1. Proc. Natl. Acad. Sci. U.S.A..

[60] Fritsch R.M., Schneider G., Saur D., Scheibel M., Schmid R.M. (2007). Translational repression of MCL-1 couples stress-induced eIF2 alpha phosphorylation to mitochondrial apoptosis initiation. J. Biol. Chem..

[61] Han J., Goldstein L.A., Gastman B.R., Rabinovitz A., Rabinowich H. (2005). Disruption of Mcl-1.Bim complex in granzyme B-mediated mitochondrial apoptosis. J. Biol. Chem..

[62] Michels J., O'Neill J.W., Dallman C.L., Mouzakiti A., Habens F., Brimmell M., Zhang K.Y., Craig R.W., Marcusson E.G., Johnson P.W. (2004). Mcl-1 is required for Akata6 B-lymphoma cell survival and is converted to a cell death molecule by efficient caspase-mediated cleavage. Oncogene.

[63] Herrant M., Jacquel A., Marchetti S., Belhacene N., Colosetti P., Luciano F., Auberger P. (2004). Cleavage of Mcl-1 by caspases impaired its ability to counteract Bim-induced apoptosis. Oncogene.

[64] Clohessy J.G., Zhuang J., Brady H.J. (2004). Characterisation of Mcl-1 cleavage during apoptosis of haematopoietic cells. Br. J. Haematol..

[65] Weng C., Li Y., Xu D., Shi Y., Tang H. (2005). Specific cleavage of Mcl-1 by caspase-3 in tumor necrosis factor-related apoptosis-inducing ligand (TRAIL)-induced apoptosis in Jurkat leukemia T cells. J. Biol. Chem..

[66] Rechsteiner M., Rogers S.W. (1996). PEST sequences and regulation by proteolysis. Trends Biochem. Sci..

[67] Kobayashi S., Lee S.H., Meng X.W., Mott J.L., Bronk S.F., Werneburg N.W., Craig R.W., Kaufmann S.H., Gores G.J. (2007). Serine 64 phosphorylation enhances the antiapoptotic function of Mcl-1. J. Biol. Chem..

[68] Domina A.M., Vrana J.A., Gregory M.A., Hann S.R., Craig R.W. (2004). MCL1 is phosphorylated in the PEST region and stabilized upon ERK activation in viable cells, and at additional sites with cytotoxic okadaic acid or taxol. Oncogene.

[69] Ding Q., Huo L., Yang J.Y., Xia W., Wei Y., Liao Y., Chang C.J., Yang Y., Lai C.C., Lee D.F. (2008). Down-regulation of myeloid cell leukemia-1 through inhibiting Erk/Pin 1 pathway by sorafenib facilitates chemosensitization in breast cancer. Cancer Res..

[70] Nifoussi S.K., Vrana J.A., Domina A.M., De Biasio A., Gui J., Gregory M.A., Hann S.R., Craig R.W. (2012). Thr 163 phosphorylation causes Mcl-1 stabilization when degradation is independent of the adjacent GSK3-targeted phosphodegron, promoting drug resistance in cancer. PloS one.

[71] Inoshita S., Takeda K., Hatai T., Terada Y., Sano M., Hata J., Umezawa A., Ichijo H. (2002). Phosphorylation and inactivation of myeloid cell leukemia 1 by JNK in response to oxidative stress. J. Biol. Chem..

[72] Kodama Y., Taura K., Miura K., Schnabl B., Osawa Y., Brenner D.A. (2009). Antiapoptotic effect of c-Jun N-terminal Kinase-1 through Mcl-1 stabilization in TNF-induced hepatocyte apoptosis. Gastroenterology.

[73] Nijhawan D., Fang M., Traer E., Zhong Q., Gao W., Du F., Wang X. (2003). Elimination of Mcl-1 is required for the initiation of apoptosis following ultraviolet irradiation. Genes Dev..

[74] Cuconati A., Mukherjee C., Perez D., White E. (2003). DNA damage response and MCL-1 destruction initiate apoptosis in adenovirus-infected cells. Genes Dev..

[75] Derouet M., Thomas L., Cross A., Moots R.J., Edwards S.W. (2004). Granulocyte macrophage colony-stimulating factor signaling and proteasome inhibition delay neutrophil apoptosis by increasing the stability of Mcl-1. J. Biol. Chem..

[76] Nencioni A., Hua F., Dillon C.P., Yokoo R., Scheiermann C., Cardone M.H., Barbieri E., Rocco I., Garuti A., Wesselborg S. (2005). Evidence for a protective role of Mcl-1 in proteasome inhibitor-induced apoptosis. Blood.

[77] Willis S.N., Chen L., Dewson G., Wei A., Naik E., Fletcher J.I., Adams J.M., Huang D.C. (2005). Proapoptotic Bak is sequestered by Mcl-1 and Bcl-xL, but not Bcl-2, until displaced by BH3-only proteins. Genes Dev..

[78] Zhong Q., Gao W., Du F., Wang X. (2005). Mule/ARF-BP1, a BH3-only E3 ubiquitin ligase, catalyzes the polyubiquitination of Mcl-1 and regulates apoptosis. Cell.

[79] Warr M.R., Acoca S., Liu Z., Germain M., Watson M., Blanchette M., Wing S.S., Shore G.C. (2005). BH3-ligand regulates access of MCL-1 to its E3 ligase. FEBS Lett..

[80] Warr M.R., Mills J.R., Nguyen M., Lemaire-Ewing S., Baardsnes J., Sun K.L., Malina A., Young J.C., Jeyaraju D.V., O'Connor-McCourt M. (2011). Mitochondrion-dependent N-terminal processing of outer membrane Mcl-1 protein removes an essential Mule/Lasu1 protein-binding site. J. Biol. Chem..

[81] Czabotar P.E., Lee E.F., van Delft M.F., Day C.L., Smith B.J., Huang D.C., Fairlie W.D., Hinds M.G., Colman P.M. (2007). Structural insights into the degradation of Mcl-1 induced by BH3 domains. Proc. Natl. Acad. Sci. U.S.A..

[82] Mei Y., Du W., Yang Y., Wu M. (2005). Puma(*)Mcl-1 interaction is not sufficient to prevent rapid degradation of Mcl-1. Oncogene.

[83] Wuilleme-Toumi S., Trichet V., Gomez-Bougie P., Gratas C., Bataille R., Amiot M. (2007). Reciprocal protection of Mcl-1 and Bim from ubiquitin-proteasome degradation. Biochem. Biophys. Res. Commun..

[84] Gomez-Bougie P., Menoret E., Juin P., Dousset C., Pellat-Deceunynck C., Amiot M. (2011). Noxa controls Mule-dependent Mcl-1 ubiquitination through the regulation of the Mcl-1/USP9X interaction. Biochem. Biophys. Res. Commun..

[85] Inoue S., Hao Z., Elia A.J., Cescon D., Zhou L., Silvester J., Snow B., Harris I.S., Sasaki M., Li W.Y. (2013). Mule/Huwe1/Arf-BP1 suppresses Ras-driven tumorigenesis by preventing c-Myc/Miz1-mediated down-regulation of p21 and p15. Genes Dev..

[86] Hao Z., Duncan G.S., Su Y.W., Li W.Y., Silvester J., Hong C., You H., Brenner D., Gorrini C., Haight J. (2012). The E3 ubiquitin ligase Mule acts through the ATM-p53 axis to maintain B lymphocyte homeostasis. J. Exp. Med..

[87] Ding Q., He X., Hsu J.M., Xia W., Chen C.T., Li L.Y., Lee D.F., Liu J.C., Zhong Q., Wang X. (2007). Degradation of Mcl-1 by beta-TrCP mediates glycogen synthase kinase 3-induced tumor suppression and chemosensitization. Mol. Cell. Biol..

[88] Inuzuka H., Shaik S., Onoyama I., Gao D., Tseng A., Maser R.S., Zhai B., Wan L., Gutierrez A., Lau A.W. (2011). SCFFBW7 regulates cellular apoptosis by targeting MCL1 for ubiquitylation and destruction. Nature.

[89] Maurer U., Charvet C., Wagman A.S., Dejardin E., Green D.R. (2006). Glycogen synthase kinase-3 regulates mitochondrial outer membrane permeabilization and apoptosis by destabilization of MCL-1. Mol. Cell.

[90] Morel C., Carlson S.M., White F.M., Davis R.J. (2009). Mcl-1 integrates the opposing actions of signaling pathways that mediate survival and apoptosis. Mol. Cell. Biol..

[91] Zhao Y., Altman B.J., Coloff J.L., Herman C.E., Jacobs S.R., Wieman H.L., Wofford J.A., Dimascio L.N., Ilkayeva O., Kelekar A. (2007). Glycogen Synthase Kinase 3{alpha} and 3{beta} Mediate a Glucose-Sensitive Antiapoptotic Signaling Pathway To Stabilize Mcl-1. Mol. Cell. Biol..

[92] Magiera M.M., Mora S., Mojsa B., Robbins I., Lassot I., Desagher S. (2013). Trim17-mediated ubiquitination and degradation of Mcl-1 initiate apoptosis in neurons. Cell Death Differ..

[93] Jope R.S., Johnson G.V. (2004). The glamour and gloom of glycogen synthase kinase-3. Trends Biochem. Sci..

[94] Ding Q., He X., Xia W., Hsu J.M., Chen C.T., Li L.Y., Lee D.F., Yang J.Y., Xie X., Liu J.C. (2007). Myeloid cell leukemia-1 inversely correlates with glycogen synthase kinase-3beta activity and associates with poor prognosis in human breast cancer. Cancer Res..

[95] Ren H., Koo J., Guan B., Yue P., Deng X., Chen M., Khuri F.R., Sun S.Y. (2013). The E3 ubiquitin ligases beta-TrCP and FBXW7 cooperatively mediates GSK3-dependent Mcl-1 degradation induced by the Akt inhibitor API-1, resulting in apoptosis. Mol Cancer.

[96] Dehan E., Bassermann F., Guardavaccaro D., Vasiliver-Shamis G., Cohen M., Lowes K.N., Dustin M., Huang D.C., Taunton J., Pagano M. (2009). betaTrCP- and Rsk1/2-mediated degradation of BimEL inhibits apoptosis. Mol. Cell.

[97] Frescas D., Pagano M. (2008). Deregulated proteolysis by the F-box proteins SKP2 and beta-TrCP: tipping the scales of cancer. Nat. Rev. Cancer.

[98] Wertz I.E., Kusam S., Lam C., Okamoto T., Sandoval W., Anderson D.J., Helgason E., Ernst J.A., Eby M., Liu J. (2011). Sensitivity to antitubulin chemotherapeutics is regulated by MCL1 and FBW7. Nature.

[99] Wang Z., Inuzuka H., Zhong J., Wan L., Fukushima H., Sarkar F.H., Wei W. (2012). Tumor suppressor functions of FBW7 in cancer development and progression. FEBS Lett..

[100] Millman S.E., Pagano M. (2011). MCL1 meets its end during mitotic arrest. EMBO Rep..

[101] Harley M.E., Allan L.A., Sanderson H.S., Clarke P.R. (2010). Phosphorylation of Mcl-1 by CDK1-cyclin B1 initiates its Cdc20-dependent destruction during mitotic arrest. EMBO J..

[102] Meroni G., Diez-Roux G. (2005). TRIM/RBCC, a novel class of 'single protein RING finger' E3 ubiquitin ligases. Bioessays.

[103] Napolitano L.M., Meroni G. (2012). TRIM family: Pleiotropy and diversification through homomultimer and heteromultimer formation. IUBMB Life.

[104] Ogawa S., Goto W., Orimo A., Hosoi T., Ouchi Y., Muramatsu M., Inoue S. (1998). Molecular cloning of a novel RING finger-B box-coiled coil (RBCC) protein, terf, expressed in the testis. Biochem. Biophys. Res. Commun..

[105] Urano T., Usui T., Takeda S., Ikeda K., Okada A., Ishida Y., Iwayanagi T., Otomo J., Ouchi Y., Inoue S. (2009). TRIM44 interacts with and stabilizes terf, a TRIM ubiquitin E3 ligase. Biochem. Biophys. Res. Commun..

[106] Endo H., Ikeda K., Urano T., Horie-Inoue K., Inoue S. (2012). Terf/TRIM17 stimulates degradation of kinetochore protein ZWINT and regulates cell proliferation. J. Biochem. (Tokyo)..

[107] Lassot I., Robbins I., Kristiansen M., Rahmeh R., Jaudon F., Magiera M.M., Mora S., Vanhille L., Lipkin A., Pettmann B. (2010). Trim17, a novel E3 ubiquitin-ligase, initiates neuronal apoptosis. Cell Death Differ..

[108] Schwickart M., Huang X., Lill J.R., Liu J., Ferrando R., French D.M., Maecker H., O'Rourke K., Bazan F., Eastham-Anderson J. (2010). Deubiquitinase USP9X stabilizes MCL1 and promotes tumour cell survival. Nature.

[109] Stewart D.P., Koss B., Bathina M., Perciavalle R.M., Bisanz K., Opferman J.T. (2010). Ubiquitin-independent degradation of antiapoptotic MCL-1. Mol. Cell. Biol..

[110] Jariel-Encontre I., Bossis G., Piechaczyk M. (2008). Ubiquitin-independent degradation of proteins by the proteasome. Biochim. Biophys. Acta.

[111] Baugh J.M., Viktorova E.G., Pilipenko E.V. (2009). Proteasomes can degrade a significant proportion of cellular proteins independent of ubiquitination. J. Mol. Biol..

